# 1,3-Bis(ethoxy­meth­yl)-1*H*-benzimidazole-2(3*H*)-thione

**DOI:** 10.1107/S1600536810013036

**Published:** 2010-04-24

**Authors:** Augusto Rivera, Alexander Mejia-Camacho, Jaime Ríos-Motta, Michal Dušek, Karla Fejfarová

**Affiliations:** a Departamento de Química, Universidad Nacional de Colombia, Bogotá, AA 14490, Colombia; b Institute of Physics, Na Slovance 2, 182 21 Praha 8, Czech Republic

## Abstract

In the structure of the title compound, C_13_H_18_N_2_O_2_S, mol­ecules are linked together by inter­molecular C—H⋯S inter­actions into one-dimensional extended chains along the *a* axis. The crystal packing is further influenced by weak C—H⋯O inter­actions.

## Related literature

For related structures, see: Odabaşoğlu *et al.* (2007 ▶). For applications and uses of benzimidazole-2-thio­nes, see: Zhang *et al.* (2001 ▶, 2007 ▶); Monforte *et al.* (2008 ▶); Mazloum *et al.* (2000 ▶); Perrin & Pagetti (1998 ▶). For chemical background on the synthesis of the title compound, see: Wang & Liu (1996 ▶, 2007 ▶); Rivera & Maldonado (2006 ▶); Rivera *et al.* (2008 ▶).
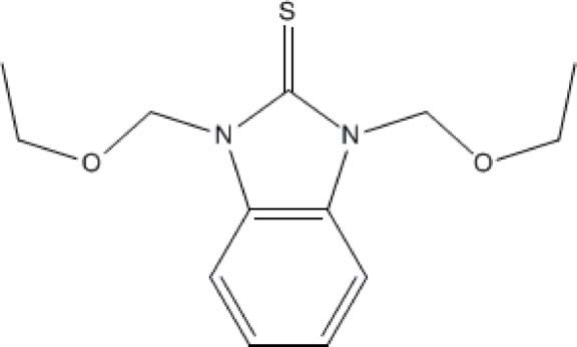


## Experimental

### 

#### Crystal data

C_13_H_18_N_2_O_2_S
*M* *_r_* = 266.4
Monoclinic, 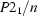

*a* = 4.7176 (2) Å
*b* = 16.0664 (6) Å
*c* = 17.5128 (6) Å
β = 96.524 (3)°
*V* = 1318.78 (9) Å^3^
*Z* = 4
Cu *K*α radiation
μ = 2.14 mm^−1^
*T* = 120 K
0.36 × 0.09 × 0.07 mm

#### Data collection

Oxford Diffraction Xcalibur diffractometer with an Atlas (Gemini ultra Cu) detector
Absorption correction: multi-scan (*CrysAlis PRO*; Oxford Diffraction, 2009 ▶) *T*_min_ = 0.239, *T*_max_ = 1.000
11526 measured reflections
2096 independent reflections
1718 reflections with *I* > 3σ(*I*)
*R*_int_ = 0.035
θ_max_ = 62.3°

#### Refinement

*R*[*F* ^2^ > 2σ(*F* ^2^)] = 0.038
*wR*(*F* ^2^) = 0.110
*S* = 2.09
2096 reflections
163 parameters
H-atom parameters constrained
Δρ_max_ = 0.40 e Å^−3^
Δρ_min_ = −0.20 e Å^−3^

### 

Data collection: *CrysAlis CCD* (Oxford Diffraction, 2009 ▶); cell refinement: *CrysAlis RED* (Oxford Diffraction, 2009 ▶); data reduction: *CrysAlis RED*; program(s) used to solve structure: *SIR2002* (Burla *et al.*, 2003 ▶); program(s) used to refine structure: *JANA2006* (Petříček *et al.*, 2006 ▶); molecular graphics: *DIAMOND* (Brandenburg & Putz, 2005 ▶); software used to prepare material for publication: *JANA2006*.

## Supplementary Material

Crystal structure: contains datablocks global, I. DOI: 10.1107/S1600536810013036/bt5233sup1.cif

Structure factors: contains datablocks I. DOI: 10.1107/S1600536810013036/bt5233Isup2.hkl

Additional supplementary materials:  crystallographic information; 3D view; checkCIF report

## Figures and Tables

**Table 1:** Hydrogen-bond geometry (Å, °)

*D*—H⋯*A*	*D*—H	H⋯*A*	*D*⋯*A*	*D*—H⋯*A*
C4—H4⋯O2^i^	0.95	2.57	3.489 (2)	158.46
C7—H7⋯O1^ii^	0.95	2.58	3.480 (2)	155.41
C12—H12*a*⋯S1^iii^	0.96	2.88	3.7915 (19)	158.58

Symmetry codes: (i) 
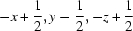
; (ii) 
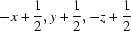
; (iii) 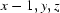.

**Fig. 1.**
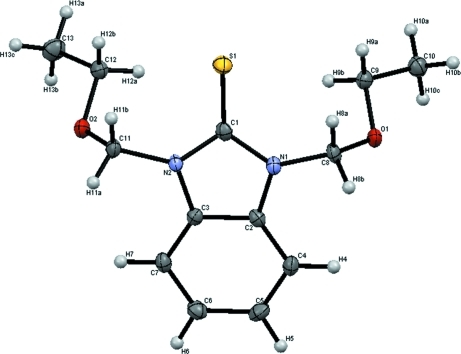
The molecular structure of the title compound showing the atom-numbering scheme, with atomic displacement ellipsoids drawn at the 50% probability level.

**Fig. 2.**
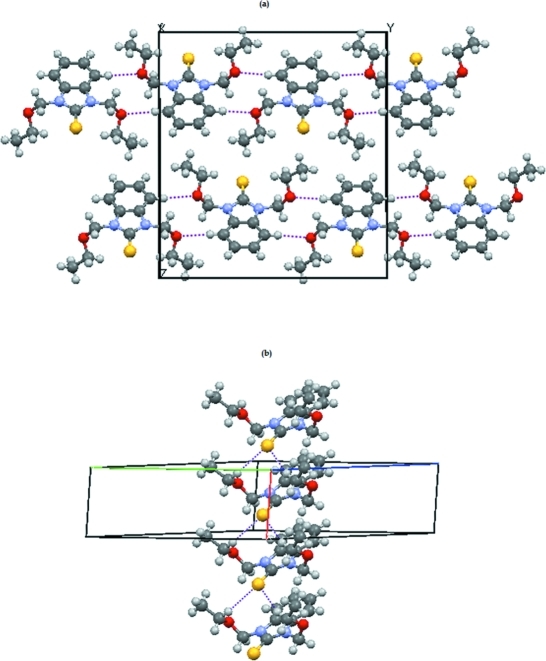
Packing diagram with two different views; hydrogen bonds drawn as dashed lines.

## supplementary crystallographic information

### Comment

Benzimidazole-2-thione and their derivatives exhibit potential applications in many areas such as: pharmacological (Zhang *et al.* 2001, 2007; Monforte *et al. *2008) and industrial (Mazloum *et al.* 2000; Perrin & Pagetti, 1998). This compound has been synthesized by reaction of *o*-phenylenediamine with carbon disulfide in presence of KOH (Wang & Liu, 2007 ) or tertiary amines (Wang & Liu,1996). Further substitution of heterocyclic system could be obtained by *N*-alkylation with an alkylating agent. As a part of our research on the structure and properties of aminals cage, we have recently started a study on the reactivity of 6*H*,13*H*-5:12,7:14-dimethanedibenzo-[*d,i*][1,3,6,8]-tetraazecine (DMDBTA) (Rivera *et al.*, 2008, Rivera & Maldonado 2006). In our recent investigation, when we carried out the reaction between DMDBTA and carbon disulfide in ethyl alcohol, the cyclic thiourea 1,3-bis(ethoxymethyl)-1,3-dihydro-2*H*-benzimidazole-2-thione was obtained and its crystal structure was determined.

The molecular structure of the title compound, a new benzimidazole-2-thione derivative, is shown in Fig. 1. The bond lengths and angles are within normal ranges and are comparable with the related structures (Odabaşoğlu *et al.*, 2007). The crystal structure is further stabilized by intermolecular C—H···S interactions which link neighbouring molecules into 1-D extended chains along the *a *axis. The interesting feature of the crystal structure is C—H···S distance (2.88 Å), which is shorter than the sum of the Van der Waals radii of S and H by 0.12 Å. A weak intermolecular C—H···O interaction helps to establish the crystal packing which link neighbouring molecules into 1-D extended chains along the *b*-axis (Fig. 2). This X-ray analysis also shows that both the C8—O1 [1.406 (2) A] and C11—O2 [1.407 (2) A] bonds appear to be shorter than the normal C—O bond-length, whereas the other C—O bond lengths are more agreement with the typical 1.45 Å. This information indicates that the shortening of these bonds suggests some degrees of double bond character.

### Experimental

A mixture of CS_2_ (0,95 mmol) and DMDBTA (0,95 mmol) in ethanol (30 ml) was stirred at room temperature for 72 hours. After completion of reaction as monitored by TLC the solvent was distilled off in vacuo. The crude residue was purified by column chromatography over silica gel (60-120 mesh), using benzene:ethyl acetate mixture (80:20) as eluent to give the title compound. A suitable single crystal (m.p. 377-379 K) of the product was formed by slow evaporation of an acetone solution at room temperature.

The NMR spectra were acquired at room temperature on a Bruker AMX 400 Advanced spectrometer. ^1^H NMR (δ, 399.9 MHz, CDCl_3_) δ: 1.18 (6*H*, t, J=6.7 Hz –CH_3_), 3.64 (4*H*, q, J= 6.7 Hz, O—CH_2-_CH_3_) 5.82 (4*H*, s, N—CH_2_—O-), 7.27 (2*H*, m). ^13^C NMR (100 MHz, CDCl_3_) δ: 15.0, 64.9, 74.3, 110.1, 123.7, 131.7, 171.8. MS (ESI): [M+H]^+ ^267.

### Refinement

All hydrogen atoms were discernible in difference Fourier maps and could be refined to reasonable geometry. According to common practice H atoms attached to C atoms were nevertheless kept in ideal positions during the refinement. The isotropic atomic displacement parameters of hydrogen atoms were evaluated as 1.2**U*_eq_ of the parent atom.

### Crystal data

**Table:**

C_13_H_18_N_2_O_2_S	*F*(000) = 568
*M**_r_* = 266.4	*D*_x_ = 1.341 Mg m^−^^3^
Monoclinic, *P*2_1_/*n*	Cu *K*α radiation, λ = 1.54184 Å
Hall symbol: -P 2yn	Cell parameters from 7933 reflections
*a* = 4.7176 (2) Å	θ = 3.7–62.4°
*b* = 16.0664 (6) Å	µ = 2.14 mm^−^^1^
*c* = 17.5128 (6) Å	*T* = 120 K
β = 96.524 (3)°	Needle, colorless
*V* = 1318.78 (9) Å^3^	0.36 × 0.09 × 0.07 mm
*Z* = 4	

### Data collection

**Table:**

Oxford diffraction Xcalibur diffractometer with an Atlas (Gemini ultra Cu) detector	2096 independent reflections
Radiation source: X-ray tube	1718 reflections with *I* > 3σ(*I*)
mirror	*R*_int_ = 0.035
Detector resolution: 10.3784 pixels mm^-1^	θ_max_ = 62.3°, θ_min_ = 3.7°
Rotation method data acquisition using ω scans	*h* = −5→5
Absorption correction: multi-scan (*CrysAlis PRO*; Oxford Diffraction, 2009)	*k* = −17→18
*T*_min_ = 0.239, *T*_max_ = 1.000	*l* = −19→19
11526 measured reflections	

### Refinement

**Table:**

Refinement on *F*^2^	72 constraints
*R*[*F*^2^ > 2σ(*F*^2^)] = 0.038	H-atom parameters constrained
*wR*(*F*^2^) = 0.110	Weighting scheme based on measured s.u.'s *w* = 1/[σ^2^(*I*) + 0.0016*I*^2^]
*S* = 2.09	(Δ/σ)_max_ = 0.001
2096 reflections	Δρ_max_ = 0.40 e Å^−^^3^
163 parameters	Δρ_min_ = −0.20 e Å^−^^3^
0 restraints	

### Special details

**Table:**

Experimental. CrysAlisPro, Oxford Diffraction Ltd., Version 1.171.33.51 (release 27-10-2009 CrysAlis171 .NET) Empirical absorption correction using spherical harmonics, implemented in SCALE3 ABSPACK scaling algorithm.
Refinement. The refinement was carried out against all reflections. The conventional *R*-factor is always based on *F*. The goodness of fit as well as the weighted *R*-factor are based on *F* and *F*^2^ for refinement carried out on *F* and *F*^2^, respectively. The threshold expression is used only for calculating *R*-factors etc. and it is not relevant to the choice of reflections for refinement.The program used for refinement, Jana2006, uses the weighting scheme based on the experimental expectations, see _refine_ls_weighting_details, that does not force *S* to be one. Therefore the values of *S* are usually larger than the ones from the SHELX program.

### Fractional atomic coordinates and isotropic or equivalent isotropic displacement parameters (Å^2^)

**Table:**

	*x*	*y*	*z*	*U*_iso_*/*U*_eq_	
S1	0.66272 (9)	0.13029 (3)	0.10850 (3)	0.01931 (18)	
O1	0.2405 (3)	−0.07593 (7)	0.17048 (7)	0.0188 (4)	
O2	0.2199 (3)	0.33197 (7)	0.16433 (7)	0.0195 (4)	
N1	0.3657 (3)	0.06085 (9)	0.21698 (8)	0.0149 (5)	
N2	0.3556 (3)	0.19758 (9)	0.21569 (8)	0.0145 (5)	
C1	0.4612 (4)	0.12960 (10)	0.18039 (10)	0.0155 (5)	
C2	0.1990 (4)	0.08518 (11)	0.27371 (10)	0.0146 (5)	
C3	0.1912 (4)	0.17210 (11)	0.27292 (10)	0.0139 (5)	
C4	0.0581 (4)	0.03918 (11)	0.32458 (10)	0.0177 (6)	
C5	−0.0933 (4)	0.08446 (12)	0.37497 (11)	0.0208 (6)	
C6	−0.0995 (4)	0.17090 (12)	0.37354 (11)	0.0206 (6)	
C7	0.0434 (4)	0.21684 (11)	0.32276 (10)	0.0175 (6)	
C8	0.4585 (4)	−0.02431 (11)	0.20549 (11)	0.0176 (6)	
C9	0.1567 (4)	−0.05799 (11)	0.09047 (10)	0.0184 (6)	
C10	−0.0375 (4)	−0.12746 (11)	0.05950 (11)	0.0226 (6)	
C11	0.4382 (4)	0.28343 (10)	0.20354 (11)	0.0175 (6)	
C12	0.1586 (4)	0.31173 (12)	0.08429 (10)	0.0202 (6)	
C13	−0.0308 (4)	0.37927 (12)	0.04732 (12)	0.0271 (7)	
H4	0.063937	−0.020541	0.325288	0.0212*	
H5	−0.194831	0.055195	0.411191	0.0249*	
H6	−0.206135	0.199806	0.408844	0.0247*	
H7	0.039587	0.276581	0.322332	0.021*	
H8a	0.615369	−0.023937	0.175068	0.0211*	
H8b	0.53383	−0.047767	0.254005	0.0211*	
H9a	0.322743	−0.056905	0.063475	0.0221*	
H9b	0.054516	−0.006229	0.085929	0.0221*	
H10a	−0.113617	−0.114992	0.007583	0.0271*	
H10b	0.068439	−0.178548	0.060477	0.0271*	
H10c	−0.191054	−0.133156	0.090688	0.0271*	
H11a	0.50122	0.308783	0.252159	0.021*	
H11b	0.602547	0.284351	0.17585	0.021*	
H12a	0.060143	0.259395	0.079039	0.0242*	
H12b	0.333216	0.31009	0.061016	0.0242*	
H13a	−0.091255	0.364675	−0.005165	0.0325*	
H13b	−0.194805	0.385461	0.07462	0.0325*	
H13c	0.07297	0.430762	0.048906	0.0325*	

### Atomic displacement parameters (Å^2^)

**Table:**

	*U*^11^	*U*^22^	*U*^33^	*U*^12^	*U*^13^	*U*^23^
S1	0.0197 (3)	0.0185 (3)	0.0207 (3)	−0.00013 (18)	0.0067 (2)	−0.00118 (18)
O1	0.0274 (7)	0.0112 (6)	0.0176 (7)	−0.0036 (5)	0.0012 (5)	−0.0003 (5)
O2	0.0273 (8)	0.0129 (7)	0.0181 (7)	0.0033 (5)	0.0022 (6)	0.0001 (5)
N1	0.0165 (8)	0.0109 (8)	0.0174 (8)	−0.0002 (6)	0.0020 (6)	−0.0006 (6)
N2	0.0164 (8)	0.0103 (7)	0.0169 (8)	0.0000 (6)	0.0025 (6)	−0.0016 (6)
C1	0.0147 (9)	0.0146 (10)	0.0164 (9)	−0.0007 (7)	−0.0020 (7)	−0.0016 (7)
C2	0.0136 (9)	0.0145 (9)	0.0155 (9)	0.0008 (7)	0.0003 (7)	−0.0027 (7)
C3	0.0128 (9)	0.0140 (9)	0.0141 (9)	−0.0018 (7)	−0.0013 (7)	0.0009 (7)
C4	0.0194 (10)	0.0124 (10)	0.0205 (10)	−0.0012 (7)	−0.0008 (8)	0.0005 (7)
C5	0.0210 (10)	0.0232 (10)	0.0179 (10)	−0.0055 (8)	0.0015 (8)	0.0052 (8)
C6	0.0211 (10)	0.0212 (10)	0.0201 (10)	0.0027 (8)	0.0051 (8)	−0.0025 (8)
C7	0.0191 (10)	0.0124 (10)	0.0205 (10)	0.0017 (7)	−0.0003 (8)	−0.0009 (7)
C8	0.0203 (10)	0.0106 (9)	0.0215 (10)	0.0029 (7)	0.0006 (8)	−0.0028 (7)
C9	0.0219 (10)	0.0148 (9)	0.0185 (9)	0.0028 (7)	0.0029 (7)	0.0016 (7)
C10	0.0255 (11)	0.0201 (11)	0.0216 (10)	0.0000 (8)	0.0003 (8)	−0.0013 (8)
C11	0.0220 (11)	0.0115 (9)	0.0186 (10)	−0.0033 (7)	0.0007 (7)	0.0005 (7)
C12	0.0244 (11)	0.0182 (10)	0.0181 (10)	−0.0043 (8)	0.0026 (8)	−0.0014 (7)
C13	0.0275 (11)	0.0282 (12)	0.0246 (11)	0.0005 (8)	−0.0011 (9)	0.0036 (8)

### Geometric parameters (Å, °)

**Table:**

S1—C1	1.6618 (19)	C6—H6	0.96
O1—C8	1.406 (2)	C7—H7	0.96
O1—C9	1.441 (2)	C8—H8a	0.96
O2—C11	1.407 (2)	C8—H8b	0.96
O2—C12	1.436 (2)	C9—C10	1.505 (3)
N1—C1	1.378 (2)	C9—H9a	0.96
N1—C2	1.392 (2)	C9—H9b	0.96
N1—C8	1.457 (2)	C10—H10a	0.96
N2—C1	1.376 (2)	C10—H10b	0.96
N2—C3	1.397 (2)	C10—H10c	0.96
N2—C11	1.456 (2)	C11—H11a	0.96
C2—C3	1.397 (2)	C11—H11b	0.96
C2—C4	1.384 (3)	C12—C13	1.504 (3)
C3—C7	1.379 (3)	C12—H12a	0.96
C4—C5	1.401 (3)	C12—H12b	0.96
C4—H4	0.96	C13—H13a	0.96
C5—C6	1.389 (3)	C13—H13b	0.96
C5—H5	0.96	C13—H13c	0.96
C6—C7	1.388 (3)		
			
C8—O1—C9	114.35 (13)	N1—C8—H8b	109.4708
C11—O2—C12	113.95 (13)	H8a—C8—H8b	105.1992
C1—N1—C2	110.36 (14)	O1—C9—C10	106.89 (14)
C1—N1—C8	124.70 (15)	O1—C9—H9a	109.4714
C2—N1—C8	124.42 (15)	O1—C9—H9b	109.4714
C1—N2—C3	110.40 (14)	C10—C9—H9a	109.4711
C1—N2—C11	124.75 (15)	C10—C9—H9b	109.4713
C3—N2—C11	124.23 (15)	H9a—C9—H9b	111.9358
S1—C1—N1	127.08 (13)	C9—C10—H10a	109.4709
S1—C1—N2	127.05 (13)	C9—C10—H10b	109.4709
N1—C1—N2	105.86 (15)	C9—C10—H10c	109.4713
N1—C2—C3	106.86 (15)	H10a—C10—H10b	109.4713
N1—C2—C4	131.39 (16)	H10a—C10—H10c	109.4718
C3—C2—C4	121.75 (16)	H10b—C10—H10c	109.4711
N2—C3—C2	106.50 (15)	O2—C11—N2	113.78 (14)
N2—C3—C7	131.54 (16)	O2—C11—H11a	109.4713
C2—C3—C7	121.95 (17)	O2—C11—H11b	109.4723
C2—C4—C5	116.41 (16)	N2—C11—H11a	109.4702
C2—C4—H4	121.7924	N2—C11—H11b	109.4712
C5—C4—H4	121.793	H11a—C11—H11b	104.7886
C4—C5—C6	121.26 (18)	O2—C12—C13	107.50 (15)
C4—C5—H5	119.3691	O2—C12—H12a	109.4709
C6—C5—H5	119.369	O2—C12—H12b	109.4714
C5—C6—C7	122.17 (18)	C13—C12—H12a	109.4711
C5—C6—H6	118.9156	C13—C12—H12b	109.4711
C7—C6—H6	118.916	H12a—C12—H12b	111.3742
C3—C7—C6	116.46 (17)	C12—C13—H13a	109.4719
C3—C7—H7	121.7704	C12—C13—H13b	109.4712
C6—C7—H7	121.771	C12—C13—H13c	109.471
O1—C8—N1	113.43 (14)	H13a—C13—H13b	109.471
O1—C8—H8a	109.4721	H13a—C13—H13c	109.4706
O1—C8—H8b	109.4715	H13b—C13—H13c	109.4717
N1—C8—H8a	109.4706		

### Hydrogen-bond geometry (Å, °)

**Table:**

*D*—H···*A*	*D*—H	H···*A*	*D*···*A*	*D*—H···*A*
C4—H4···O2^i^	0.95	2.57	3.489 (2)	158.46
C7—H7···O1^ii^	0.95	2.58	3.480 (2)	155.41
C8—H8a···S1	0.95	2.75	3.2180 (19)	110.16
C11—H11b···S1	0.95	2.77	3.2166 (19)	109.21
C12—H12a···S1^iii^	0.96	2.88	3.7915 (19)	158.58

Symmetry codes: (i) −*x*+1/2, *y*−1/2, −*z*+1/2; (ii) −*x*+1/2, *y*+1/2, −*z*+1/2; (iii) *x*−1, *y*, *z*.
